# Bone Incorporation of a Poly (L-Lactide-Co-D, L-Lactide) Internal Fixation Device in a Rat’s Tibia: Microtomographic, Confocal LASER, and Histomorphometric Analysis

**DOI:** 10.3390/biology13070471

**Published:** 2024-06-26

**Authors:** Harrisson Lucho Mamani-Valeriano, Nelson Padilha Silva, Heloisa Helena Nímia, Maísa Pereira-Silva, Maria Eduarda de Freitas Santana Oliveira, Letícia Gabriella de Souza Rodrigues, Paulo Matheus Honda Tavares, Henrique Hadad, Laís Kawamata de Jesus, Ana Flávia Piquera Santos, Débora de Barros Barbosa, Pier Paolo Poli, Carlo Maiorana, Paulo Sergio Perri de Carvalho, Roberta Okamoto, Francisley Ávila Souza

**Affiliations:** 1 Department of Diagnosis and Surgery, School of Dentistry, São Paulo State University (UNESP), Araçatuba 16010-380, SP, Brazil; harrisson.mamani@unesp.br (H.L.M.-V.); maisa.silva@unesp.br (M.P.-S.); freitas.santana@unesp.br (M.E.d.F.S.O.); leticia.rodrigues@unesp.br (L.G.d.S.R.); paulo.matheus@unesp.br (P.M.H.T.); henriquehadad@gmail.com (H.H.); lais.kawamata@unesp.br (L.K.d.J.); ana.f.santos@unesp.br (A.F.P.S.); 2 Department of Dental Materials and Prothesis, School of Dentistry, São Paulo State University (UNESP), Araçatuba 16010-380, SP, Brazil; h.nimia@unesp.br (H.H.N.); debora.b.barbosa@unesp.br (D.d.B.B.); 3 Implant Center for Edentulism and Jawbone Atrophies, Maxillofacial Surgery and Odontostomatology Unit, Fondazione IRCCS Cà Granda Ospedale Maggiore Policlinico, 20122 Milan, Italy; pierpaolo.poli@unimi.it (P.P.P.); carlo.maiorana@unimi.it (C.M.); 4 Department of Biomedical, Surgical and Dental Sciences, University of Milan, 20122 Milan, Italy; 5 Implant Dentistry Postgraduate Program, São Leopoldo Mandic School of Dentistry and Research Center, Campinas 13045-755, SP, Brazil; paulo.perri@unesp.br; 6 Department of Basic Sciences, School of Dentistry, São Paulo State University (UNESP), Araçatuba 16015-253, SP, Brazil; roberta.okamoto@unesp.br

**Keywords:** absorbable implants, bone regeneration, bone screws, osseointegration, X-ray microtomography

## Abstract

**Simple Summary:**

Poly (L-lactide-co-D, L-lactide) and PLDLLA have mechanical properties such as biodegradability, biocompatibility, thermoplastic capacity, and physical properties. In addition, their use eliminates the need for removal in subsequent surgeries and reduces the discomfort and risks associated with additional procedures. Given these advantages, a national company is currently developing an internal fixation device manufactured for PLDLLA, and it is essential to evaluate its bone incorporation process. The PLDLLA fixation device tested in this animal study was biocompatible and facilitated the formation of new bone around the device. Therefore, to avoid a second surgical procedure, this device is a highly effective option for those seeking a reliable and safe solution for bone fixation procedures.

**Abstract:**

This study evaluated the bone incorporation process of a screw-shaped internal fixation device made of poly (L-lactide-co-D, L-lactide) (PLDLLA). Thirty-two male Wistar rats received 32 fixation devices (2 mm × 6 mm) randomly assigned to either the right or left tibia and one implant in each animal. After 7, 14, 28, and 42 days, the rats were euthanized and the specimens were subjected to microtomographic computed tomography (microCT) and histomorphometric analyses to evaluate bone interface contact (BIC%) and new bone formation (NBF%) in cortical and cancellous bone areas. The animals euthanized on days 28 and 42 were treated with calcein and alizarin red, and confocal LASER microscopy was performed to determine the mineral apposition rate (MAR). Micro-CT revealed a higher percentage of bone volume (*p* < 0.006), trabecular separation (*p* < 0.001), and BIC in the cortical (*p* < 0.001) and cancellous (*p* = 0.003) areas at 28 and 42 days than at 7 and 14 days. The cortical NBF at 42 days was greater than that at 7 and 14 days (*p* = 0.022). No statistically significant differences were observed in cancellous NBF or MAR at 28 and 42 days. Based on these results, it can be seen that the PLDLLA internal fixation device is biocompatible and allows new bone formation around the screw thread.

## 1. Introduction

Various biodegradable implants, such as screws, pins, and plates, are commercially available. Implants are composed of biodegradable polymers with specific characteristics [1]. They are commonly used in dentistry and medicine, particularly in procedures such as guided bone regeneration (GBR); thermal filling; and oral, maxillofacial, and orthopedic surgeries [2,3,4]. The most common resorbable biomaterials include polyglycolic acid (PGA), polylactic acid (PLA), and polydioxanone (PDS) [5,6,7]. These materials offer a sufficient fixation strength and can undergo manipulation, hydrolysis, and biphasic metabolic processes [8,9]. As a result, the implants experience a reduction in molecular weight, followed by a decrease in mechanical resistance, and finally, in mass, allowing the material to be gradually reabsorbed by the body over 1–5 years. They are then replaced with newly formed bone [9,10].

Various forms of polylactic acid (PLA), such as poly l-lactic acid (PLLA) and poly d-lactic acid (PDLA), exhibit unique properties. PLLA is known for its high crystallinity and stability, whereas PDLA degrades more rapidly but has excellent biocompatibility [11]. PLLA, which has been widely used since the 1990s, is valued for its stability and slower reabsorption, whereas PDLA, although it degrades more quickly, is highly biocompatible [9,12]. When combined to form poly DL-lactic acid (PDLLA), the resulting amorphous polymer exhibits favorable mechanical, thermal, rheological, and biological properties, as well as radiotransparency [9]. PDLLA has been extensively used in the manufacturing of osteosynthetic materials [13]. PLDLLA is a copolymer of poly-(L, DL-lactide) in varying proportions, commonly 70/30, offering a balance of properties [14]. Previous studies demonstrated promising results using PLDLLA membranes to promote bone formation through osteoconduction and osteoinduction in cranial vault defects [15].

Polymer manipulation results in the formation of resorbable products in the body. These products, which contain substances such as lactic and glycolic acids which are found naturally in the body, are safely metabolized or excreted and pose no harm. They meet the standard specifications for nontoxicity [16]. These components offer advantages such as biodegradability, elimination of the need for additional surgeries for removal, and supporting tissue regeneration. However, they may exhibit a lower mechanical strength than non-resorbable materials such as titanium alloys and can be metabolized prematurely in certain clinical contexts [17,18]. Manufacturing involves polymerization techniques that combine L-lactic acid and D, L-lactic acid monomers to form the desired polymers [19].

PLDLLA has unique properties, such as biodegradability, biocompatibility, and thermoplastic capacity. These properties eliminate the need for removal during subsequent surgeries and reduce the discomfort and risks associated with additional procedures [6,20,21]. Thus, it is an excellent option for surgeries requiring membrane fixation in GBR. Therefore, a national company is developing an internal fixation device made from PLDLLA. The device aims to promote the stabilization of membranes and grafts in GBR as well as to reduce and fix bone fractures in fixation systems and osteosynthesis.

Therefore, it is essential to evaluate the process of incorporation into bone tissue during the developmental phase. The objective of this study was to evaluate the bone incorporation of a PLDLLA internal fixation device in the tibia of rats using microtomography, laser confocal microscopy, and histomorphometric analysis.

## 2. Materials and Methods

### 2.1. Study Design and Ethics

This experimental study was performed according to the Ethical Principles for Animal Experimentation adopted by the Brazilian Animal Experimentation Board (COBEA) and was approved by the Ethical Committee for Animal Use (CEUA) of the Araçatuba School of Dentistry, UNESP (CEUA 0335-2022). The project was designed in accordance with Animal Research: Reporting of In Vivo Experiments (ARRIVE) guidelines [22]. Thirty-two male Wistar Albinus rats, 3 months old, weighing 350 g and 450 g, respectively, were included in this study. The rats were housed in ventilated cages with a 12/12-h light/dark cycle and were fed and watered ad libitum.

To determine the power of the sample, a level of significance of 5% (with a standard deviation of 2%), and with a test power of 80%, with alpha being defined as 0.05, was set. Eight rats per group were needed for the purpose of comparing the different groups. There were an estimated number of 8 animals for each period group [23,24,25,26,27]. The animals randomly received an internal fixation device (screws) made of PLDLLA (Baumer, Mogi Mirim, São Paulo, Brazil) in their right or left tibia. The rats were euthanized after 7, 14, 28, and 42 days for further analysis, and no groups were excluded.

### 2.2. PLDLLA Device

The tested device consisted of a fixation screw made of a poly (L-lactide-co-D,L-lactide) polymer with a length of 6 mm and diameter of 2 mm (Baumer, Mogi Mirim, São Paulo, Brazil). Each fixation device was carefully delivered into a sterilized capsule by the manufacturer. This biomaterial (internal fixation device) is included in the list of biomaterial experimental projects under development by the Baumer Company from Brazil.

### 2.3. Surgical Procedure

All surgical procedures were conducted in accordance with the methodology described in previously published studies [23,24,25,26,27]. Prior to surgery, the animals were fasted for 12 h, and general anesthesia was induced by intramuscular administration of 50 mg/kg ketamine (Vetaset–Fort Dodge Saúde Animal Ltda., São Paulo, Brazil) and 5 mg/kg xylazine (Dopaser–Laboratório Calier do Brazil Ltda., São Paulo, Brazil). Complementary anesthesia was administered locally at the surgical site with mepivacaine at a dose of 0.3 mL/kg (scandicaine 2% with adrenaline 1:100,000, Septogon, Saint Maur des Fossé, France). The surgical access was made on the anterior region of the randomly chosen tibia, which was shaved and preoperatively cleansed with Polyvinyl Pyrrolidone Iodine (PVP-I) degermante 10% (Riodeine Degermante, Rioquímica, São José do Rio Preto).

**Figure 1:**
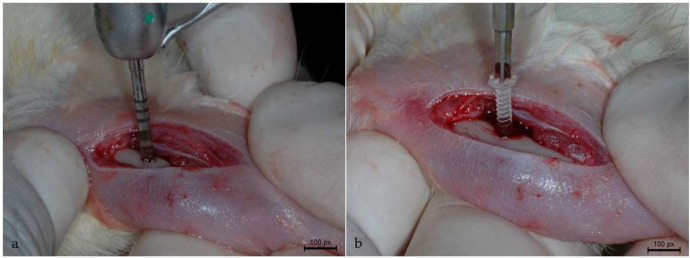
Experimental surgery: (**a**) Osteotomy; (**b**) installation of device fixation (screw).

A dermoperiosteal incision was made on the anterior margin of the tibia starting 5 mm below the tibiofemoral joint and measuring approximately 1 cm in length. After the incision was made, the flap was displaced with consequent exposure of the tibia. Osteotomy was performed by means of a 1.6 mm diameter and 6 mm length helical drill (Emfils, Novo Colosso, Itu, Brazil) mounted in a contra-angle handpiece with a 20:1 reduction (Kavo do Brasil, Joinville, Brazil), coupled to an electric motor (Kavo do Brasil, Joinville, Brazil), at a speed of 1200 rpm (Figure 1a), under constant irrigation of 0.9% physiological solution (Darrow, Rio de Janeiro, Brazil).

After preparing the surgical site, the internal devices were installed using a digital cross-key (Baumer, Mogi Mirim, São Paulo, Brazil). To avoid the risk of fracture, a fixation device was used to stabilize the PLDLLA internal screws in each animal (Figure 1b). The muscle was sutured using an absorbable polyglactin 910 thread (Vicryl 5-0, Ethicon, Johnson Prod., São José dos Campos, Brazil), whereas the cutaneous suture was made with a non-absorbable nylon monofilament (Ethicon 5-0, Johnson, São José dos Campos, Brazil). Post-operative antisepsis procedure was performed using 10% polyvinylpyrrolidone iodide. After the surgery, the animals received a single dose of intramuscular administration of Pentabiotic 0.1 mL/kg (Fort Dodge Saúde Animal Ltda., São Paulo, Brazil) and sodium dipyrone 1 mg/kg/day (Ariston Indústrias Químicas e Farmacêuticas Ltda., São Paulo, Brazil).

### 2.4. Fluorochrome Application

Fluorochrome was administered to the animals during the 28-day and 42-day study periods to analyze the mineral apposition rate, according to previous studies [23,28,29]. Animals allocated to the 28-day group received an intramuscular injection of calcein 20 mg/kg 14 days after screw installation, followed by alizarin red 30 mg/kg 10 days later. Animals assigned to the 42-day period received intramuscular calcein and alizarin red at 24 and 34 post-operative days, respectively.

### 2.5. Euthanasia and Sample Extraction

Prior to euthanasia, all the animals underwent a twelve-hour fasting period. The animals were sedated with an intramuscular injection of ketamine hydrochloride at a dose of 50 mg/kg (Vetaset—Fort Dodge Animal Health Ltd., Campinas, SP, Brazil) and xylazine hydrochloride at a dose of 5 mg/kg (Dopaser, Laboratórios Calier do Brasil Ltd., Osasco, SP, Brazil). Following sedation, the animals were euthanized using a lethal dose of sodium thiopental (150 mg/kg; Tiopentax, Cristália Ltd., Itapira, SP, Brazil). The tibias of the animals containing the tested fixation device were collected after euthanasia, and the excess soft tissue was removed, followed by a reduction of 10 mm on each side around the fixation device. The tibias were then placed in a 10% buffered formalin solution (Analytical Reagents, Dinâmica Odonto-Hospitalar Ltd., Catanduva, SP, Brazil) for 24 h, after which they were subjected to a bath in running water for additional 24 h. The tissues were stored in 70% alcohol until micro-computed tomography (CT) analysis.

### 2.6. Micro-computed Tomography Analysis (Micro-CT)

Micro-computed tomography (micro-CT) analysis was performed according to the methodology outlined in a previous study [30]. The samples were scanned using a Skyscan micro-tomography device (SkyScan 1272 Bruker MicroCT, Aatselaar, Belgium, 2003) with 6 µm thick slices, 90 kV, and 111 μA, and an Al filter of 0.5 mm + Cu 0.038. The rotation step was set to 0.5 mm, the pixel size to 2.016 × 1.344 μm, and the acquisition time to 1 h and 15 min. Images obtained by projecting X-rays onto the samples were stored and reconstituted. The area of interest was determined using NRecon software (SkyScan, 2011; Version 1.6.6.0) with a smoothing of 1, artifact ring correction of 8, correction of Beam Hardening of 24%, and an image conversion range, which varied from 0.0 to 0.14.

The images were analyzed in a linear 2D reconstructed view using Data Viewer software (SkyScan, Version 1.4.4 64-bit) and observed in three planes (transverse, longitudinal, and sagittal) and transaxial views. The CTAnalyzer—CTAn (2003-11SkyScan, 2012 erMicroCT Version 1.12.4.0) software was used to determine the study region (ROI-region of interest) using the “Round” tool, corresponding to the cortical and cancellous areas surrounding the internal fixation device. All pieces were standardized and 3D and volume measurements were obtained. The following parameters were evaluated from the obtained images: bone volume (BV), percentage of bone volume/trabecular volume (BV/TV), trabecular thickness (Tb.Th), trabecular separation (TB.Sp), and number of trabeculae (Tb.N).

### 2.7. Laboratory Processing

Following micro-CT analysis, the 32 tibias of the animals underwent a series of steps for further analysis, as described in prior studies [31,32,33,34]. The tibias were dehydrated using a gradual sequence of alcohols at varying concentrations (60%, 70%, 80%, 90%, and 100%). Once fully dehydrated, the pieces were immersed in a mixture of 100% alcohol and Techno Vit^®^ light-curing resin (Munich, Germany, Heraeus Kulzer GmbH Division Technik Philipp-Reis-Str. 8/13 D-61273 Wehrheim) at varying concentrations until only the resin remained in the immersion medium. The pieces were then embedded in Technovit^®^ resin, light-cured, and subjected to a processing protocol for cutting and grinding of calcified pieces. The pieces containing the bone tissue and device were cut at a central point of the fixation device, using an Exakt-type microtome (Cutting System, Apparatebau, Gmbh, Hamburg, Germany), resulting in pieces in the coronal plane with a section thickness of approximately 60 μm.

### 2.8. LASER Confocal Microscopy

The confocal LASER microscope Leica CTR 4000 CS SPE (Leica Microsystems, Heidelberg, Germany) captured images of sections of the pieces at 28 and 42 days using a 10× magnification at the Laboratory of Confocal Fluorescence Microscopy at the Araraquara School of Dentistry (UNESP). Thus, images of calcein and alizarin red fluorochromes were obtained separately (old bone/new bone) and reconstructed. Superimposition of fluorochromes was performed to assess bone turnover based on the mineral apposition rate (MAR).

The precipitation area of fluorochromes (calcein/alizarin) was measured using the ImageJ 1.54i image analyzer software (Image Processing and Analysis Software, Toronto, ON, Canada), and the analysis of mineral bone apposition used the “Straight Line or freehands” tool. Ten measurements were drawn, extending from the external margin of the calcein towards the external margin of the alizarin. The value obtained was divided by 10, representing the number of days between injections of the two fluorochromes analyzed to determine the MAR [35].

### 2.9. Histomorphometric Analysis

After confocal microscopy, all the slides were stained with Stevenel’s blue and acidic fuchsin. Images were captured using an optical microscope (DM750, Leica Microsystems Vertrieb GmbH, Munich, Germany) at a magnification of 20× and a digital camera (Leica Microsystems, ICC50E Camera Module, Germany) with a resolution of 5.0 megapixels. Histomorphometric analyses were performed using the ImageJ image analysis software (Image Processing and Analysis Software, Toronto, ON, Canada).

To obtain measurements, the ruler was calibrated and the “Segmented Line” or “Freehand” tool was used to measure the linear extension of the fixation device/bone contact (bone interface contact—BIC) and new bone formation (NBF). The measurements were standardized and the results obtained in micrometers were converted to percentages for both the cortical and cancellous areas.

### 2.10. Statistical Analysis

Statistical analyses were performed using SigmaPlot version 12. A homogeneity test (Shapiro–Wilk) was initially conducted to verify the data distribution [36]. Kruskal–Wallis one-way analysis was used to analyze bone volume (BV), percentage of bone volume/trabecular volume (BV/TV), and new bone formation (NBF) in the cancellous area. One-way analysis of variance (ANOVA) was used to analyze trabecular thickness (Tb.Th), trabecular Number (Tb.N), trabecular separation (Tb.Sp), bone interface contact (BIC) in cortical and cancellous tissues, and new bone formation (NBF) in the cortical area. Differences between means were verified using Tukey’s post hoc test for multiple comparisons. The mineral apposition rate (MAR) distribution was checked using a homogeneity test (Shapiro–Wilk test) and *t*-tests were performed.

## 3. Results

### 3.1. Micro-CT Analysis

The micro-tomographic analysis showed statistically significant differences between periods in the parameters of BV (*p* = 0.006), BV/TV (*p* = 0.006), Tb.Th (*p* = 0.008), Tb.N (*p* < 0.001), and Tb.Sp (*p* < 0.001).

#### 3.1.1. Percentage of Bone Volume/Trabecular Volume (BV/TV)

**Figure 2:**
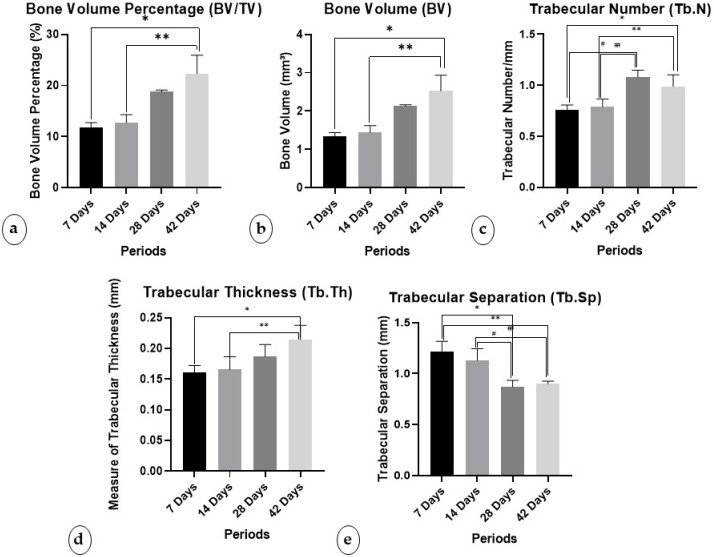
Micro-CT analysis: (**a**) percentage of bone volume; (**b**) bone volume; (**c**) trabecular number; (**d**) trabecular thickness; (**e**) trabecular separation. * denotes statistical difference between 7 days and 42 days; ** denotes statistical difference between 14 days and 42 days; # denotes statistical difference between 7 days and 28 days; ## denotes statistical difference between 14 days and 42 days.

The BV/TV percentages were high in the 42-day period (22.30 ± 3.62), followed by the 28-day period (18.82 ± 0.28). In this parameter, a statistically significant difference was found between the periods of 42, 7, and 14 days (*p* < 0.050). (Figure 2a).

#### 3.1.2. Bone Volume (BV)

In this parameter, a statistically significant difference was found between values obtained at 42 days, 7 days, and 14 days. (*p* < 0.050) The average BVs were higher in the 42-day period (2.52 ± 0.41), followed by the 28-day period (2.13 ± 0.03) (Figure 2b).

#### 3.1.3. Trabecular Number (Tb.N)

Regarding Tb.N, the average was highest in the 42-day period (0.988 ± 0.114), followed by the 28-day period (1.081 ± 0.066). Statistical differences were found at 28 days compared to 7 days (*p* < 0.001) and 14 days (*p* = 0.001), and at 42 days compared to 7 days (*p* = 0.008) and 14 days (*p* = 0.020) (Figure 2c).

#### 3.1.4. Trabecular Thickness (Tb.Th)

The statistical difference was evident between the periods of 42 days and 7 days (*p* = 0.010) and 14 days (*p* = 0.018). And the highest mean trabecular thickness in the 42-day period (0.214 ± 0.0234), followed by the 28-day period (0.187 ± 0.0197).

#### 3.1.5. Trabecular Separation (Tb.Sp)

Regarding Tb.Sp, the 7-day period (1.218 ± 0.0994 mm) and 14-day period (1.126 ± 0.118 mm) presented higher means. Statistical differences were found in the periods of 7 days compared to 28 days (*p* < 0.001) and 42 days (*p* = 0.001), and 14 days compared to 28 days (*p* = 0.005) and 42 days (*p* = 0.012). (Figure 2e)

#### 3.1.6. Qualitative Analysis

**Figure 3:**
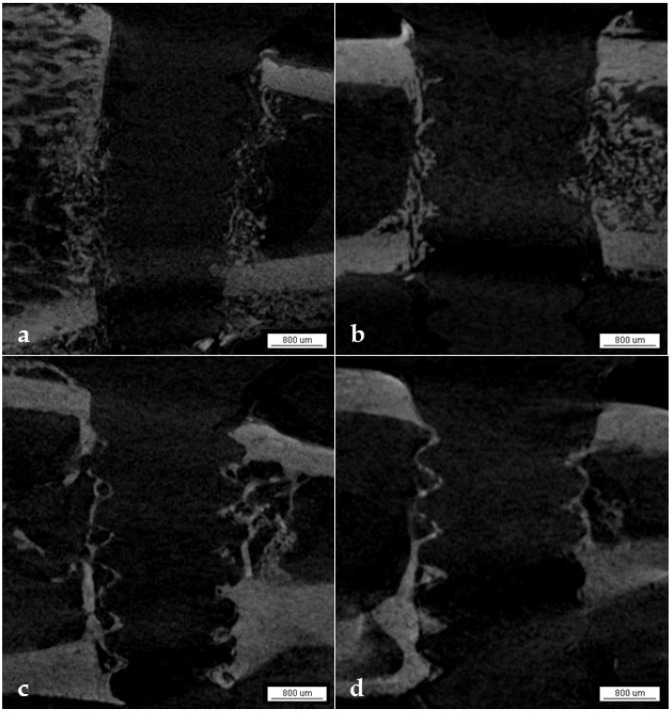
Micro-CT 3D reconstruction (**a**–**d**): gradual incorporation of PLDLLA fixation devices into bone tissue over post-operative periods of 7, 14, 28, and 42 days through the progressive deposition of minerals around the threads of the device, respectively.

In the micro-CT analysis, we observed the gradual incorporation of PLDLLA fixation devices into the bone tissue over periods of 7, 14, 28, and 42 days through the progressive deposition of minerals around the threads of the device (Figure 3a–d). At seven days, a shadow from the fixation device was visible in the tibia, with minimal mineral deposition (Figure 3a). Subsequently, on day 14, a more significant mineral deposition was observed around the threads of the device (Figure 3b), suggesting the possibility of bone tissue formation. After 28 days, the threads of the device presented a more defined shape (Figure 3c), indicating further mineral deposition. Finally, at 42 days, the most significant amount of mineral deposition was observed along the entire length of the fixation device (Figure 3d), as evidenced by the images of well-defined threads.

### 3.2. LASER Confocal Microscopy

#### Mineral Apposition Rate (MAR)

**Figure 4:**
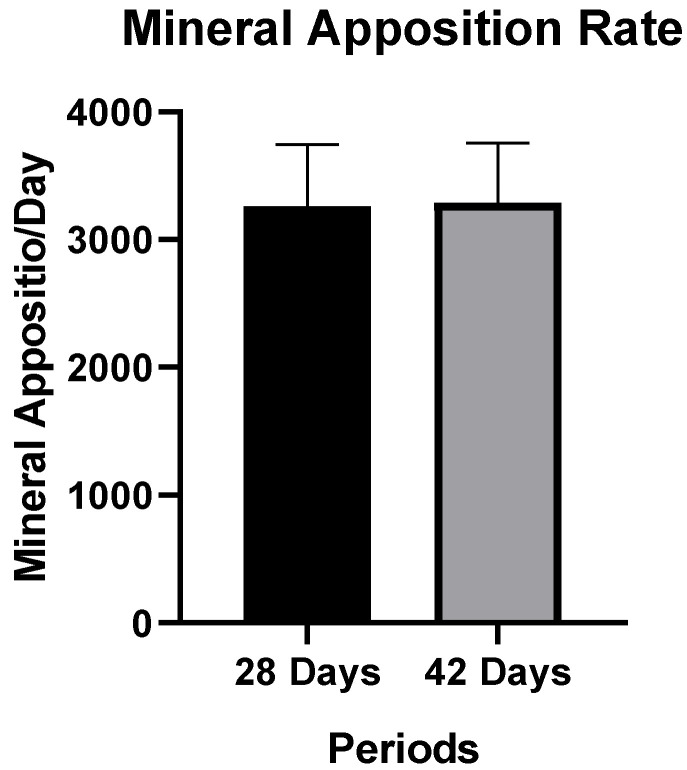
Mineral apposition rate (MAR, µm) calculated through fluorochrome during the 28-day and 42-day post-operative periods.

**Figure 5:**
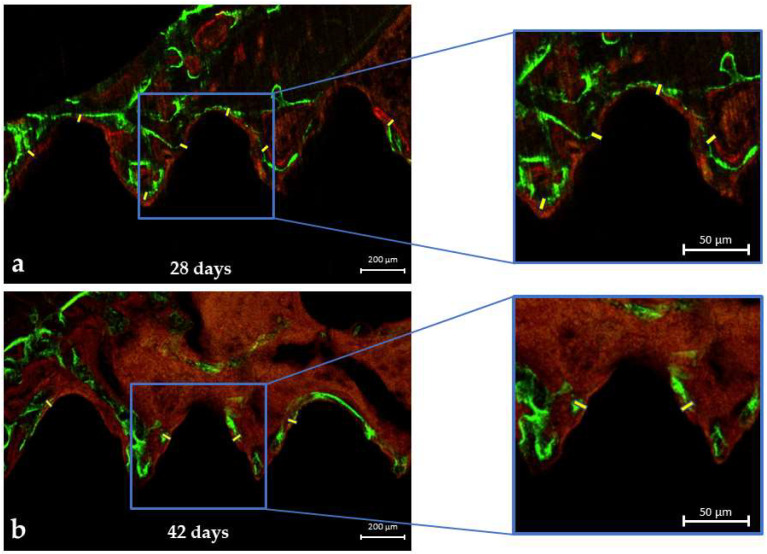
The confocal LASER microscopy analysis: (**a**,**b**) Fluorochrome images at the beginning of calcein precipitation up to alizarin red precipitation and divided per 28 and 42 days, respectively (interval of analysis)—original magnification ×10 and ×20.

The confocal LASER microscopy analysis showed no significant differences in the MAR parameter between the 28-day and 42-day periods (*p* = 0.925). However, the authors pointed out that the average MAR values at 42 days (3287.6 ± 468.8) were slightly higher than the average values for the 28-day period (3258.3 ± 481.7) (Figure 4). These results imply that there could be a gradual formation of bone tissue around the fixation device at 28 (Figure 5a) and 42 days (Figure 5b).

### 3.3. Histomorphometric Analysis

The histometric analysis demonstrated statistical differences between periods in the parameters of fixation device/bone contact (BIC) cortical area (*p* < 0.001), BIC cancellous area (*p* = 0.003), and new bone formation (NBF) cortical area (*p* = 0.022). However, no statistical difference was observed in the parameter of NBF in the cancellous area (*p* = 0.781).

#### 3.3.1. Fixation Device/Bone Contact (BIC%)

##### Cortical Area

**Figure 6:**
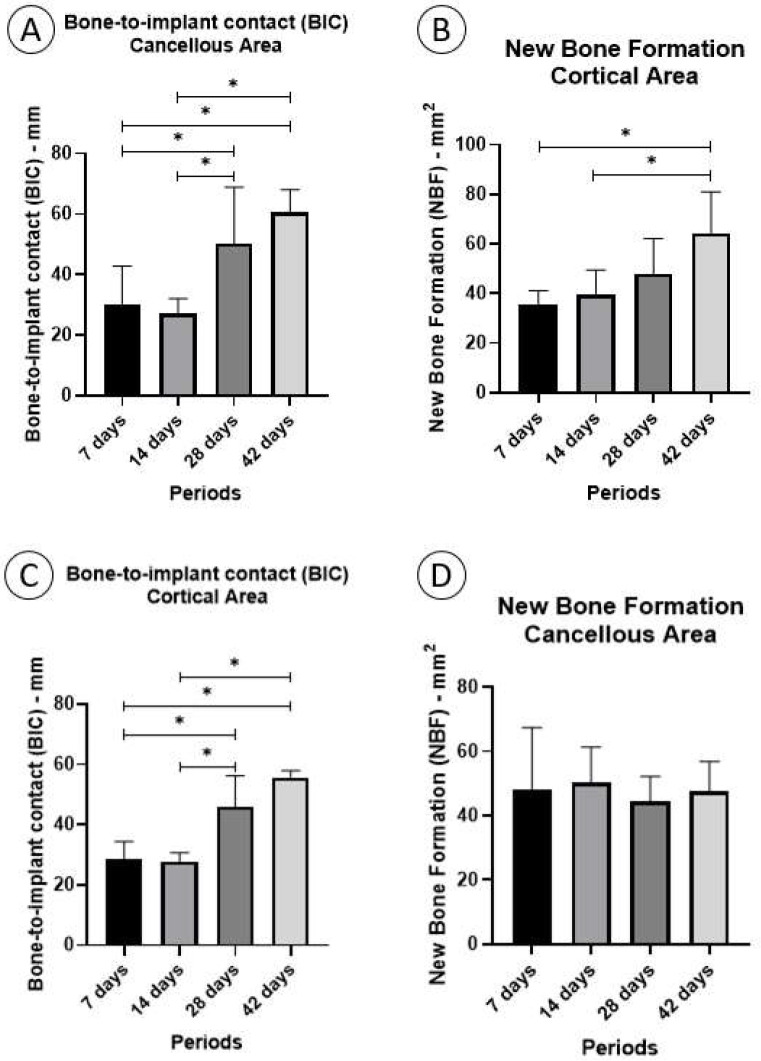
The histometric analysis: (**A**,**B**) bone-to-implant area and new bone area, respectively, in the cortical area. (**C**,**D**) Bone-to-implant area and new bone area, respectively, in the cancellous area (original magnification ×10 and ×20). * denotes statistical difference.

In the cortical area, the mean BIC% was higher in the 42-day period (55.78 ± 2.214), followed by the 28-day period (45.94 ± 10.38). Furthermore, a significant difference was noted between the 28-day period and 7 days (*p* = 0.0006) and 14 days (*p* = 0.0004). Additionally, a significant difference was found at 42 days compared with 7 days (*p* < 0.0001) and 14 days (*p* < 0.0001) (Figure 6A).

#### 3.3.2. Cancellous Area

The average BIC% in the cancellous area was higher in the 42-day period (60.56 ± 7.458) followed by the 28-day period (50.22 ± 18.76). A significant difference was observed at 28 days compared to 7 days (*p* = 0.0498) and 14 days (*p* = 0.0197). Furthermore, a significant difference was found in the period of 42 days compared to 7 days (*p* = 0.0013) and 14 days (*p* = 0.0005) (Figure 6B).

#### 3.3.3. New Bone Formation (NBF%)

##### Cortical Area

The average NBF% in the cortical area was found to be highest during the 42-day period (64.33 ± 16.61), followed by the 28-day period (47.83 ± 14.31). Furthermore, statistical differences were observed when comparing the 42-day period to the 7-day period (*p* = 0.0031) and 14-day period (*p* = 0.0118) (Figure 6C).

#### 3.3.4. Cancellous Area

In the cancellous area, the average NBF% was found to be highest during the 14-day period (50.15 ± 11.17), followed by the 7-day period (48.10 ± 19.25). However, no statistically significant differences were observed between the different time periods (Figure 6D).

#### 3.3.5. Qualitative Descriptive Analysis

**Figure 7:**
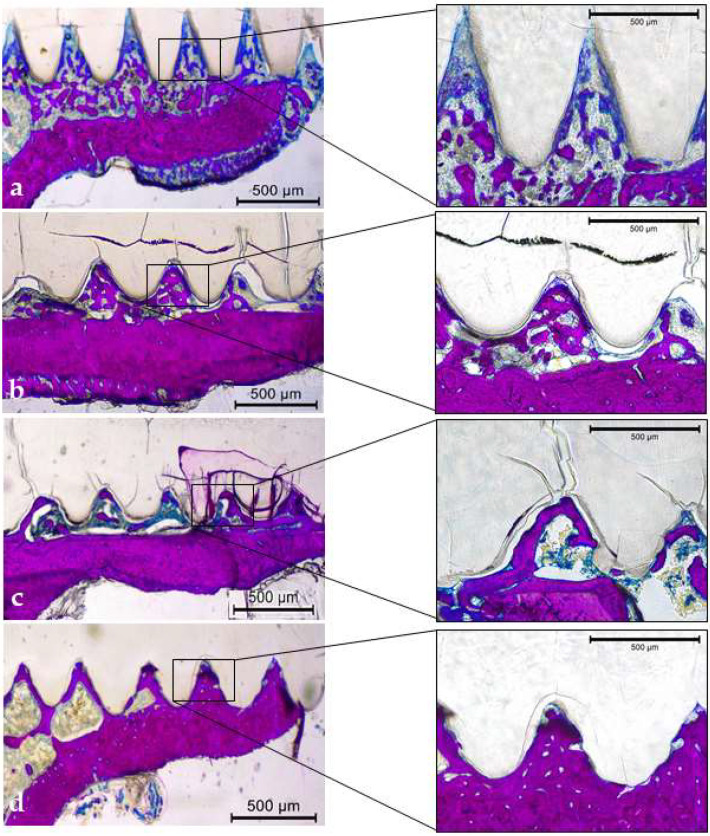
Histological sections of the mineralized superior cortical and medular areas (Stevenel blue and acid fuchsin, original magnification ×2.5 and ×20). (**a**–**d**): internal fixation device in the periods of the 7, 14, 28 and 42 days, respectively.

The PLDLLA fixation device remained intact and was gradually incorporated into the bone tissue for periods of 7, 14, 28, and 42 days without any significant degradation (Figure 7a–d). During the 7- and 14-day periods, the fixation devices remained intact without any signs of degradation. There was a considerable quantity of bone trabeculae with large cancellous spaces on day 7 (Figure 7a) and progressive bone mineralization on day 14 (Figure 7b). At 28 d, there was a significant presence of mineralized bone tissue, in which a larger number of bone trabeculae were observed (Figure 7c). After 42 days, completely mineralized bone tissue was formed between the threads (Figure 7d).

## 4. Discussion

This study investigated the incorporation of PLDLLA bioabsorbable fixation devices in the form of screws into rat tibias. However, although the fixation devices demonstrated gradual incorporation throughout the analysis period (7, 14, 28, and 42 days), no degradation of the device was observed. In the micro-CT analysis, aspects such as the percentage of bone volume (BV/TV), bone volume (BV), trabecular number (Tb.N), trabecular thickness (Tb.Th), and trabecular separation (Tb.Sp) were analyzed. Gradual deposition around the fixation device was observed during the analysis. The results achieved in the micro-CT analysis corroborate the results of the histological analysis in which parameters such as fixation device/bone contact (BIC%) and new bone formation (NBF%) presented the dynamics of gradual bone healing and the incorporation of fixation devices during the periods analyzed. The same findings were observed in the LASER confocal microscopy analysis, where the mineral apposition rate (MAR) increased from 28 to 42 days postoperatively, but without statistically significant differences. Therefore, through these analyses, we confirmed that there was a gradual incorporation of fixation devices in the form of PLDLLA screws into the bone tissue.

PLDLLA and poli (L-lactídeo-co-D, L-lactídeo) have been widely studied because of their unique properties. The combination of the two copolymers, PLLA (poli-L-lactídeo) and PDLA (poli-D-lactídeo), results in a polymer with beneficial properties, taking advantage of both [37,38]. This has led to its application in GBR and traumatology. Their unique combination of biodegradability, biocompatibility, and adaptability makes them highly attractive for use in various medical interventions [39,40,41,42].

Some orthopedic studies have investigated the incorporation and degradation of PLDLLA devices; however, in another animal model, the devices exhibited active degradation within nine months [42]. Like this study, other studies highlighted biocompatibility around the device or thread, allowing progressive bone formation [38,42]. In the microstructural analysis in the present study, by comparing the periods, we observed progressive mineral bone deposition. The bone volume percentage (BV/TV), like bone volume (BV), indicates the progression of mineral bone deposition, which refers to bone formation. However, in the analysis of the qualitative images, we see that the turns of the fixation devices (screw threads) showed dense bone when compared between days 28 and 42 and days 7 and 14. Elsewhere, no degradation in the linear extension of the fixation device was shown in the results, leading the authors to believe that its mechanical properties last for an extended period, equal to six months, given the rat’s metabolism.

A previous in vitro study examined the strength and durability of PLDLLA posts, revealing superior strength compared with PLDLLA+BTCP posts, thereby increasing their resilience [43]. Another study evaluating PLDLLA and PLA cages aligned with our findings and indicated delayed degradation. However, this study found that PLDLLA posts showed a decrease in resistance after three months compared to PLA, accompanied by the presence of microcracks [44]. In our study, a mineralized histological analysis of sections at 14 and 28 days on some slides revealed images compatible with the presence of microcracks. However, we cannot definitively attribute these microcracks to the resistance of the device or correlate them with the processing of the slices, as these images may simply be an artifact of the technique. PLDLLA resistance has been studied in the field of oral and maxillofacial surgery, presenting favorable results in orthognathic surgery. A previous study assessed the stability of PLDLLA compared to titanium fixation and concluded that PDLLA is reliable for mandibular surgeries with sagittal ramus osteotomy [45]. In addition to their resistance to fixation in trauma or orthognathic surgery, resorbable materials have been used in pediatric patients, reconstructive surgeries, and GBR [12,46,47,48,49].

In reconstructive surgeries, such as bone grafts and GBR, fixation of the grafts or membrane may be necessary, and resorbable materials may be an option. Surgery to remove metallic materials can cause tissue trauma because of the need to detach soft tissues [50]. In the present study, we found that, at 28 and 42 days, mineral apposition was stable, and at 42 days, the BIC was greater in both areas (cortical and cancellous areas), in line with previously published studies [51,52,53]. The results demonstrated the stability of the devices in the bone, demonstrating that the fixation device was incorporated. On micro-CT, trabecular thickness (Tb.Th) and trabecular separation (Tb.Sp) were inversely proportional, showing greater bone density and formation after 42 days. All these results led to the use of PLDLLA devices for graft and membrane fixation, and even for fixation fractures in traumatology using the leg screw technique.

Understanding the degradation of these fixation devices is important for their use in clinical applications such as implantology and traumatology. Previous studies have shown that the time required for the complete degradation of polymers ranges from one to five years [49,54,55,56]. This study aimed to analyze PLDLLA, which degrades faster when combined with PLLA (crystalline and faster reabsorption) and PDLA (less crystalline and faster reabsorption). Previous studies using PLDLLA screws to treat hand carpal fractures indicated a final degradation time of 12 to 14 months, as observed by radiographic examinations, suggesting complete degradation [57,58]. Although this study did not specifically investigate the degradation processes, it was evident from this preclinical in vivo model that PLDLLA did not degrade within 42 days in rats, which is equivalent to approximately six months in humans.

Based on the degradation timeline outlined in this study, along with support from the existing literature, the PLDLLA device can be deemed suitable for surgeries and clinical applications in which rapid degradation is not imperative. However, in procedures, such as block grafts requiring implant installation, the potential residue of PLDLLA must be considered. The 42-day study period, equivalent to six months in human terms, holds particular significance for operations in this domain. Nevertheless, in traumatology cases, such as the leg screw technique and even in membrane fixation in guided bone regeneration (GBR), as long as it does not interfere with areas requiring dental implant rehabilitation, the device could be considered to have excellent clinical safety.

The radiopacity of internal fixation devices is essential for their visualization in imaging tests, such as X-rays and tomography. This feature allows the devices to appear clearly on images, providing several clinical advantages: post-operative monitoring, assessment of osseous integration, identification of complications (device dislocation or mechanical failures), planning of reinterventions, and improved communication between healthcare professionals and patients [59]. Therefore, we hope that the manufacturers of this internal fixation device will increase its radiopacity in order to promote better visualization of the device.

Therefore, based on the findings of the present study, it is evident that PLDLLA did not show signs of bone degradation but showed gradual bone incorporation in the periods analyzed in this study (7, 14, 28, and 42 days). However, the existing literature suggests that degradation may occur after 12 months. Therefore, additional investigations on the initial and longer phases of degradation, acquisition of more detailed biomechanical results, and topographic characterization are required to comprehensively understand the behavior of PLDLLA.

## 5. Conclusions

In conclusion, the present study revealed that PLDLLA showed gradual incorporation during the periods analyzed (7, 14, 28, and 42 days). Therefore, to fully understand the behavior of PLDLLA, future investigations must delve deeper into the initial and prolonged degradation phases, incorporating detailed biomechanical assessments and topographic characterizations.

## Data Availability

The original contributions presented in the study are included in the article, further inquiries can be directed to the corresponding authors.
